# Antimicrobial activities of widely consumed herbal teas, alone or in combination with antibiotics: an *in vitro* study

**DOI:** 10.7717/peerj.3467

**Published:** 2017-07-26

**Authors:** Mayram Hacioglu, Sibel Dosler, Ayse Seher Birteksoz Tan, Gulten Otuk

**Affiliations:** 1 Department of Pharmaceutical Microbiology, Istanbul University, Faculty of Pharmacy, Istanbul, Turkey

**Keywords:** Checkerboard, Combination, Herbal tea, Time kill curve, Antimicrobial activity

## Abstract

**Background:**

Because of increasing antibiotic resistance, herbal teas are the most popular natural alternatives for the treatment of infectious diseases, and are currently gaining more importance. We examined the antimicrobial activities of 31 herbal teas both alone and in combination with antibiotics or antifungals against some standard and clinical isolates of *Pseudomonas aeruginosa*, *Acinetobacter baumannii*, *Escherichia coli*,* Klebsiella pneumoniae*, *Enterococcus faecalis*, methicillin susceptible/resistant *Staphylococcus aureus* and *Candida albicans*.

**Methods:**

The antimicrobial activities of the teas were determined by using the disk diffusion and microbroth dilution methods, and the combination studies were examined by using the microbroth checkerboard and the time killing curve methods.

**Results:**

Rosehip, rosehip bag, pomegranate blossom, thyme, wormwood, mint, echinacea bag, cinnamon, black, and green teas were active against most of the studied microorganisms. In the combination studies, we characterized all the expected effects (synergistic, additive, and antagonistic) between the teas and the antimicrobials. While synergy was observed more frequently between ampicillin, ampicillin-sulbactam, or nystatine, and the various tea combinations, most of the effects between the ciprofloxacin, erythromycin, cefuroxime, or amikacin and various tea combinations, particularly rosehip, rosehip bag, and pomegranate blossom teas, were antagonistic. The results of the time kill curve analyses showed that none of the herbal teas were bactericidal in their usage concentrations; however, in combination with antibiotics they showed some bactericidal effect.

**Discussion:**

Some herbal teas, particularly rosehip and pomegranate blossom should be avoided because of their antagonistic interactions with some antibiotics during the course of antibiotic treatment or they should be consumed alone for their antimicrobial activities.

## Introduction

Although antibiotics are the major drugs used for the treatment of infectious diseases, in recent years, antibiotic resistance has been increasing, and is becoming a serious problem in infection control (Akova, 2016). Some microorganisms may develop resistance to a single antimicrobial agent while others, named “multidrug-resistant (MDR)” strains, are resistant to several agents. Infections caused by these strains often fail to respond to standard treatment and generate a greater risk of death due to the spread of the resistance to other microorganisms (Giamarellou, 2010, Martis, Leroy & Blanc, 2014). In some cases, MDR microorganisms, which are called “pan-resistant organisms”, have become resistant to all the available antibiotics and cannot be treated with any single antibiotic alone (Khosravi & Mohammadian, 2016). The failure of the existing antibiotics to control infections makes it crucial to find alternative agents with new mechanisms of action. One such novel therapeutic strategy involves the use of natural antimicrobial compounds such as plant-derived products such as spices, essential oils, the extracts or the consumption of herbal teas alone or in combination with antibiotics.

Herbal teas, besides their beverage properties, are used for the treatment of human diseases worldwide (Dubick, 1986). Green and black teas, which are consumed by over two-thirds of the world’s population, are the most popular beverages next to water. Approximately 4.5 million tons of tea is produced and consumed yearly, and the largest producers are the Republic of China, India, Kenya, Sri Lanka, and Turkey (Bansal et al., 2013). The tea that originates from the leaves of the plant *Camellia sinensis (L)* Kuntze exists in four main types according to its harvesting and processing: white, green, black, and oolong. As a beverage, tea is commonly prepared by infusing the *C. sinensis* leaves in hot water. These leaves contain approximately 2,000 different phytochemicals such as phenolic compounds, methyl-xanthines, carbohydrates, proteins, free amino acids, L-ascorbic and other organic acids, volatile compounds, lipids, carotenoids, chlorophylls, minerals, and trace elements (Moderno, Carvalho & Silva, 2009).

Polyphenols are the most important constituents of tea leaves because of their higher relative abundance and bioactive properties (Moderno, Carvalho & Silva, 2009). Fresh green tea leaves are rich in monomeric flavanols known as catechins (Bansal et al., 2013). The most abundant and biologically active catechin is epigallocatechin-3-gallate (EGCG), and the other catechin derivatives are (−)-epicatechin-3-gallate, (−)-epigallocatechin, (−)-epicatechin, (+)-catechin, (+)-gallocatechin, and (−)-gallocatechin-3-gallate (Moderno, Carvalho & Silva, 2009). Tea and its components contain many health-promoting abilities such as protection from cardiovascular diseases, the control of obesity and diabetes, and have anticarcinogenic, antiaging, antihistaminic, antiarthritic, anti-inflammatory, antibacterial, antifungal, and antiviral effects (Patel, 2005).

Although the studies on other herbal teas or components are limited, there is extensive literature suggesting the health benefits of consuming teas prepared from *C. sinensis*. In particular, the antimicrobial activities of catechins against multidrug resistant clinical isolates of *Acinetobacter baumannii*, *Stenotrophomonas maltophilia*, enterohemorrhagic *Escherichia coli*, methicillin-resistant *Staphylococcus aureus* (MRSA), *Mycobacterium tuberculosis*, and *Candida sp.* have been demonstrated (Anand, Kaul & Sharma, 2006; Gordon & Wareham, 2010; Hu et al., 2002; Isogai et al., 2001; Osterburg et al., 2009; Park et al., 2011). Other herbs such as peppermint, chamomile, sage, thyme, and cinnamon also have antimicrobial activities and other health benefits (McKay & Blumberg, 2006a; McKay & Blumberg, 2006b; Peng et al., 2010; Shan et al., 2007). In the present study, we examined the antimicrobial activities of 31 herbal teas alone and in combination with antibiotics or antifungals against both standard and clinical isolates of *Pseudomonas aeruginosa*, *A. baumannii*, *E. coli*, *Klebsiella pneumoniae*, *Enterococcus faecalis*, methicillin-susceptible *S. aureus* (MSSA), MRSA, and *Candida albicans*, which can cause serious nosocomial or community-acquired infections (Kunz & Brook, 2010; Rosenthal et al., 2012; Maltezou & Giamarellou, 2006; Suleyman & Alangaden, 2016).

## Materials and Methods

### Microorganisms

A total eight clinical isolates consisting of *P. aeruginosa*, *A. baumannii*, *E. coli*, *K. pneumoniae*, *E. faecalis*, MSSA, MRSA, and *C. albicans*, obtained from specimens submitte d to the Clinical Microbiology Laboratories of Istanbul University Istanbul Faculty of Medicine, were used. Isolates were identified with Vitek 2 (BioMerieux, Marcy l’Etoile, France) and verified with API test kits (BioMerieux, Marcy l’Etoile, France). *P. aeruginosa* ATCC 27853, *A. baumannii* ATCC 19606, *E. coli* ATCC 25922, *K. pneumoniae* ATCC 4352, *E. faecalis* ATCC 29212, MSSA ATCC 29213, MRSA ATCC 43300, and *C. albicans* ATCC 10231 were used as standard strains in the study.

### Teas

Aqueous tea infusions of the following teas were prepared by adding 100 ml of boiling water to 10 g of dried leaves of green, black, thyme, linden, lemon balm, hibiscus, wormwood, rosemary, nettle, chamomile, bay, yarrow, eucalyptus, lavender, mint, rosehip, pomegranate blossom, galangal, orange, sage, cinnamon, ginger, herb bennet, and echinacea teas or bags of green, black, linden, chamomile, rosehip, sage, and echinacea teas. After 30 min of infusion, the teas were filtered through 0.40- and 0.22-µm filters. These 10% tea infusions were aliquoted and stored at −20 °C (Peng et al., 2010). All the teas were purchased from domestic markets and herbalists.

### Antibiotics and antifungals

Antimicrobials were kindly provided by their respective manufacturers, such as ampicillin, ceftazidime, cefuroxime, and amikacin from I.E. Ulagay Ilac; erythromycin, ciprofloxacin, linezolid, ampicillin–sulbactam, doxycycline, and fluconazole from Kocak Pharma Ilac; whereas itraconazole and nystatine were purchased from Sigma (Sigma, St. Louis, MO, USA). The stock solutions from the dry powders were prepared at a concentration of 1280 mg/L for the antifungals and 5120 mg/L for the antibiotics according to Clinical and Laboratory Standards Institute (CLSI) recommendations (2006, 2012, 2014). They were filtered for sterilization and stored frozen at −80 °C for up to six months.

### Media

Mueller-Hinton broth (MHB; Difco Laboratories, Detroit, Mich., USA) and Roswell Park Memorial Institute 1640 medium (RPMI) supplemented with L-glutamine and buffered with morpholine propanesulfonic acid (Sigma, St. Louis, MO, USA) were used for all the experiments as recommended by CLSI (2006, 2012, 2014). Plates of Tryptic soy agar and Sabouraud dextrose agar (Difco Laboratories, Detroit, MI, USA) were used for the colony counts.

### Antimicrobial activity

The antimicrobial activities of the teas were primarily assessed by using CLSI (2014) disc diffusion method. The minimum inhibitory concentrations (MIC) of the teas that had an antimicrobial activity, which was observed from disc diffusion tests, were determined by using the microdilution technique, as described by CLSI (2006, 2012). Serial two-fold dilutions ranging from 128 to 0.06 mg/L for ampicillin; 64 to 0.03 mg/L for erythromycin, linezolid, ampicillin–sulbactam, cefuroxime, amikacin, ceftazidime, and doxycycline; and 32 to 0.015 mg/L for ciprofloxacin were prepared in MHB and antifungals were prepared in RPMI medium. Each well was inoculated with the overnight cultures of the bacteria and fungi in a final concentrations of 1 ×10^6^ and 1 ×10^3^ colony forming units/ml (cfu) respectively. The trays were covered and placed in plastic bags to prevent evaporation, and then incubated at 37 °C 24 h for bacteria and 48 h for yeast. The sterility control for media without antimicrobials and growth controls of microorganisms were also included. The MIC was defined as the lowest concentration of the antimicrobials producing complete inhibition of visible growth, as described by CLSI (2006). For antifungals, the lowest concentration that inhibited any visible growth at 48 h was used as a MIC for nystatine whereas the significant reduction in turbidity at 530 nm compared with the control was used for fluconazole and itraconazole (CLSI, 2012). Experiments were performed in duplicates; when the results were different in both experiments, we made another test for final result confirmation.

### Determination of fractional inhibitory concentration index (FICI)

The interactions between the teas which present the MIC via microdilution method, against at least one strain as shown in Table 1, and the antimicrobials were tested by using the microbroth checkerboard technique (Pillai, Moellering Jr & Eliopoulos, 2005). Each microtiter well, containing the mixture of teas and antimicrobials in different final concentrations, ranging from 2 × MIC to 1/8 × MIC was inoculated with fresh cultures. After incubation at 37 °C for 18–20 h, the following formulas were used to calculate the FIC index: FIC_A_ = (MIC_A_ in combination)/(MIC_A_ alone), FIC_B_ = (MIC_B_ in combination)/(MIC_B_ alone), and the FIC index = FIC_A_ + FIC_B_. The combination value was derived from the highest dilution of the antimicrobial combination that permitted no visible growth. With this method, a FICI of ≤0.5 was considered synergistic, of >0.5–4 was considered to be additive, and of >4.0 was considered to be antagonistic (Odds, 2003). The experiments were performed in duplicates, when the results were different in both experiments, we made another test for final result confirmation.

**Table 1:** The MIC values of herbal teas against standard and clinical bacterial and yeast strains tested (%).

	MIC (%)														
	T	W	M	R	PB	BT	GT	O	C	RB	BTB	GTB	SB	MB	EB
**Standard strains**															
*S.aureus* ATCC 43300	0.31	0.62	–	2.5	2.5	0.31	0.07	–	–	2.5	–	0.31	–	–	–
*S.aureus* ATCC 29213	–	–	–	2.5	2.5	0.31	0.07	–	–	2.5	–	0.15	–	–	–
*E.faecalis* ATCC 29212	–	–	–	2.5	1.25	–	–	–	–	2.5	–	–	–	–	–
*E.coli* ATCC 25922	–	–	–	2.5	1.25	–	–	–	–	2.5	–	–	–	–	–
*K.pneumoniae* ATCC 4352	–	–	–	2.5	1.25	–	–	–	–	–	–	–	–	–	–
*P.aeruginosa* ATCC 27853	–	–	–	2.5	1.25	–	–	–	–	2.5	1.25	–	–	–	–
*A.baumannii* ATCC 19606	–	–	–	2.5	2.5	–	–	–	–	2.5	1.25	–	–	–	–
*C.albicans* ATCC 10231	–	–	–	–	–	0.15	0.07	–	–	–	–	–	–	–	–
**Clinical isolates**															
MRSA	0.62	1.25	0.62	1.25	1.25	0.62	0.15	0.31	–	2.5	0.31	0.15	0.62	0.62	–
MSSA	0.62	0.62	0.31	1.25	1.25	0.31	0.07	0.31	–	2.5	0.31	0.07	0.62	0.62	0.62
*E.faecalis*	–	–	–	1.25	0.62	–	–	–	–	1.25	–	–	–	–	–
*E.coli*	–	–	–	2.5	2.5	–	–	–	–	2.5	–	–	–	–	–
*K.pneumoniae*	–	–	–	2.5	2.5	–	–	–	–	2.5	–	–	–	–	–
*P.aeruginosa*	–	–	–	1.25	1.25	–	1.25	–	–	2.5	2.5	–	–	–	–
*A.baumanii*	–	–	–	1.25	1.25	–	0.31	–	–	1.25	0.62	0.62	–	–	–
*C.albicans*	–	–	–	–	–	–	–	–	2.5	–	–	–	–	–	–

**Notes.**

T thyme W wormwood M mint R rosehip PB pomegranate blossom BT black tea GT green tea O orengo C cinnamon RB rosehip bag BTB black tea bag GTB green tea bag, SB sage bag MB mint bag EB echinacea bag MRSA methicillin resistant *S. aureus* MSSA methicillin susceptible *S. aureus* (–) Not determined

### Time kill assays

In order to observe the dynamic picture of killing kinetics of the tea extracts which were significantly synergistic or antagonistic with antibiotics in checkerboard assays, the Time killing curve (TKC) method was performed according to the National Committee for Clinical Laboratory Standards (NCCLS, 1999), by testing the 1 × MICs against clinical strains. The TKCs were constructed by plotting the mean colony counts (log cfu/ml) of tea extracts alone and in combination with antibiotics, versus time. The bacterial suspensions of six different clinical isolates were incubated with antimicrobials or their combinations at 37 °C with gentle shaking, and the viable bacterial counts were performed after 0, 2, 4, 7, and 24 h of incubation. For that, one milliliter of the suspension was withdrawn and serially diluted with a sterile saline solution. Then, 100 µl of the bacterial suspensions or dilutions were spotted on the TSA plates, and the cfu was determined after the overnight incubation of the plates at 37 °C. An antibiotic-free control was included for each strain. The lowest limit of the detection for the time kill assays was 1 log cfu/ml. The antibiotic carry-over was controlled by the inhibition of the colonial growth at the side of the initial streak according to the NCCLS guidelines. The results were interpreted by the effect of the combination in comparison with that of the most active agent alone. Synergy and antagonism were defined as a 2 log cfu/ml decrease and increase, respectively, in the colony count at 24 h. The bactericidal activity was defined as a ≥3 log cfu/ml decrease from the initial inoculum (NCCLS, 1999).

## Results

### Susceptibility assays

Of the 31 teas (24 different herbs and seven bag teas), as shown in Table S1, only 15 teas showed inhibition zones against one or more microorganisms in the disk diffusion assays, while the others: linden, lemon balm, hibiscus, rosemary, nettle, chamomile, bay, yarrow, eucalyptus, lavender, galangal, orange, sage, ginger, herb bennet, and echinacea teas did not show any activity. The MIC values of the teas that were active in the disk diffusion test, along with the antibiotic and antifungal activities against clinical and standard strains of the bacteria and fungi are summarized in Tables 1 and 2. According to these results, the clinical isolates were more sensitive to teas than the standard strains. Rosehip, rosehip bag, and pomegranate blossom were the most effective teas against bacteria. Thyme, wormwood, mint, black, and green teas were highly effective against *S. aureus*. Moreover, echinacea bag and cinnamon teas were active against the clinical strains of *S. aureus* and *C. albicans* respectively.

**Table 2:** The MIC values of antibiotics and antifungals against standard and clinical bacterial and yeast strains tested (µg/ml).

Microorganisms	MIC (µg/ml)											
	ERY	CIP	AMP	LZD	SAM	CXM	AMK	CAZ	DOX	FLU	ITRA	NYS
**Standard strains**												
*S.aureus* ATCC 43300	–	1	–	2	–	–	–	–	–	–	–	–
*S.aureus* ATCC 29213	0.25	1	0.25	–	–	–	–	–	–	–	–	–
*E.faecalis* ATCC 29212	–	1	2	2	–	–	–	–	–	–	–	–
*E.coli* ATCC 25922	–	0.015	–	–	4	2	–	–	–	–	–	–
*K.pneumoniae* ATCC 4352	–	0.015	–	–	1	0.25	–	–	–	–	–	–
*P.aeruginosa* ATCC 27853	–	0.25	–	–	–	–	2	1	–	–	–	–
*A.baumannii* ATCC 19606	–	1	–	–	2	–	–	–	0.0625	–	–	–
*C.albicans* ATCC 10231	–	–	–	–	–	–	–	–	–	1	0.25	2
**Clinical isolates**												
MRSA	–	32	–	2	–	–	–	–	–	–	–	–
MSSA	0.25	0.5	128	–	–	–	–	–	–	–	–	–
*E.faecalis*	–	4	4	4	–	–	–	–	–	–	–	–
*E.coli*	–	0.015	–	–	16	0.5	–	–	–	–	–	–
*K.pneumoniae*	–	0.03	–	–	4	2	–	–	–	–	–	–
*P.aeruginosa*	–	0.25	–	–	–	–	4	1	–	–	–	–
*A.baumanii*	–	16	–	–	64	–	–	–	8	–	–	–
*C.albicans*	–	–	–	–	–	–	–	–	–	0.25	0.25	2

**Notes.**

ERY erythromycin CIP ciprofloxacin AMP ampicillin LZD linezolid SAM ampicillinsulbactam CXM cefuroxime AMK amikacin CAZ ceftazidime DOX doxycycline FLU fluconazole ITRA itraconazole NYS nystatine MRSA methicillin resistant *S. aureus* MSSA methicillin susceptible *S. aureus* (–) Not determined

### Checkerboard assay

The results of the combination studies performed using the microbroth checkerboard technique against the clinical and standard strains are shown in Tables 3 and 4. With a FICI of ≤0.5 as the borderline, synergistic interactions were observed between especially ampicillin and almost all tea combinations against MSSA followed by some ampicillin–sulbactam combinations against *E. coli*, or *A. baumannii*. Moreover, with a FICI of >4 as the borderline, antagonistic effects were observed particularly between rosehip, pomegranate, or rosehip bag teas, and ciprofloxacin against all tested strains followed by cefuroxime against *E. coli, K. pneumoniae* and *P. aeruginosa*, erythromycin, ampicillin–sulbactam, amikacin, or doxycycline against each one or two strains. There were no antagonist interactions between the teas and the antifungals tested.

**Table 3:** The FIC indexes of herbal tea and antibiotic combinations against Gram positive bacteria and *C. albicans*.

	MSSA			MRSA		*E. faecalis*			*C. albicans*		
Herbal teas+	ERY	CIP	AMP	*CIP*	*LZD*	*CIP*	LZD	AMP	FLU	ITRA	NYS
***Clinical isolates***											
R	5	9	0.6	*9*	*2*	*9*	0.6	1.1	–	–	–
PB	5	9	0.6	*9*	*2*	*5*	1	0.5	–	–	–
BT	1	4	0.3	*2*	*2*	*–*	–	–	–	–	–
GT	2	2	0.1	*0.7*	*1*	*–*	–	–	–	–	–
RB	≥9	9	0.3	*9*	*1*	*1.1*	2	2	–	–	–
GTB	2	2	0.1	*2*	*1*	*–*	–	–	–	–	–
T	1	0.6	0.5	*1*	*0.7*	*–*	–	–	–	–	–
W	2	0.6	0.5	*0.7*	*0.7*	*–*	–	–	–	–	–
M	0.6	2	0.7	*1*	*1*	*–*	–	–	–	–	–
SB	1	0.6	0.7	*2*	*1*	*–*	–	–	–	–	–
MB	0.6	3	0.1	*1*	*0.6*	*–*	–	–	–	–	–
EB	1	2.2	0.1	*–*	*–*	*–*	–	–	–	–	–
BTB	3	2	0.5	*0.7*	*0.6*	*–*	–	–	–	–	–
O	0.6	1.5	0.7	*0.7*	*0.6*	*–*	–	–	–	–	–
C	–	–	–	*–*	*–*	*–*	–	–	0.7	0.7	0.7
***Standard strains***											
R	5	5	1	*8*	*0.6*	*5*	2	0.7	–	–	–
PB	5	5	2	*9*	*1*	*9*	1	0.7	–	–	–
BT	0.7	5	0.7	*2*	*0.7*	*–*	–	–	0.7	0.7	0.5
GT	2	2	1	*2*	*0.7*	*–*	–	–	0.7	0.6	0.3
RB	5	5	2	*9*	*2*	*1.7*	2	0.7	–	–	–
GTB	0.75	2	1	*3*	*0.7*	*–*	–	–	–	–	–
T	–	–	–	*2*	*0.7*	*–*	–	–	–	–	–
W	–	–	–	*3*	*0.7*	*–*	–	–	–	–	–
C	–	–	–	*–*	*–*	*–*	–	–	0.7	0.7	1.1

**Notes.**

R rosehip PB pomegranate blossom BT black tea GT green tea RB rosehip bag GTB green tea bag T thyme W wormwood M mint SB sage bag MB mint bag EB echinacea bag BTB black tea bag O orengo C cinnamon ERY erythromycin CIP ciprofloxacin AMP ampicillin LZD linezolid FLU fluconazole ITRA itraconazole NYS nystatine (–) Not determined

**Table 4:** The FIC indexes of herbal tea and antibiotic combinations against Gram negative bacteria.

	*E. coli*			*P. aeruginosa*			*A. baumannii*			*K. pneumoniae*		
Herbal teas+	CXM	SAM	CIP	CIP	AMK	CAZ	SAM	CIP	DOX	CXM	SAM	CİP
***Clinical isolates***												
R	9	2	≥9	5	≥5	2	0.7	9	0.7	5	9	9
PB	3	0.7	≥8	5	5	2	0.7	≥8	0.7	9	3	9
GT	–	–	–	≥9	2	2	1	1	1	–	–	–
RB	1.5	0.3	≥9	5	≥5	1.5	0.5	≥8	0.7	≥4	2	≥8
GTB	–	–	–	–	–	–	1	5	3	–	–	–
BTB	–	–	–	9	≥5	1.5	0.5	5	2	–	–	–
***Standard strains***												
R	5	0.7	5	5	5	2	0.7	9	5	5	3	5
PB	5	1	5	9	9		2	9	3	5	3	5
GT	–	–	–	5	0.7	1	2	2	1.5	–	–	–
RB	5	1	5	5	9	2	2	9	5	–	–	–
GTB	–	–	–	–	–	–	–	–	–	–	–	–
BTB	–	–	–	5	5	1	2	5	5	–	–	–

**Notes.**

R rosehip PB pomegranate blossom GT green tea RB rosehip bag GTB green tea bag BTB black tea bag BT black tea CIP ciprofloxacin SAM ampicillin-sulbactam CXM cefuroxime AMK amikacin CAZ ceftazidime DOX doxycycline FLU fluconazole ITRA itraconazole NYS nystatine (–) Not determined

### Time kill assays

**Figure 1:**
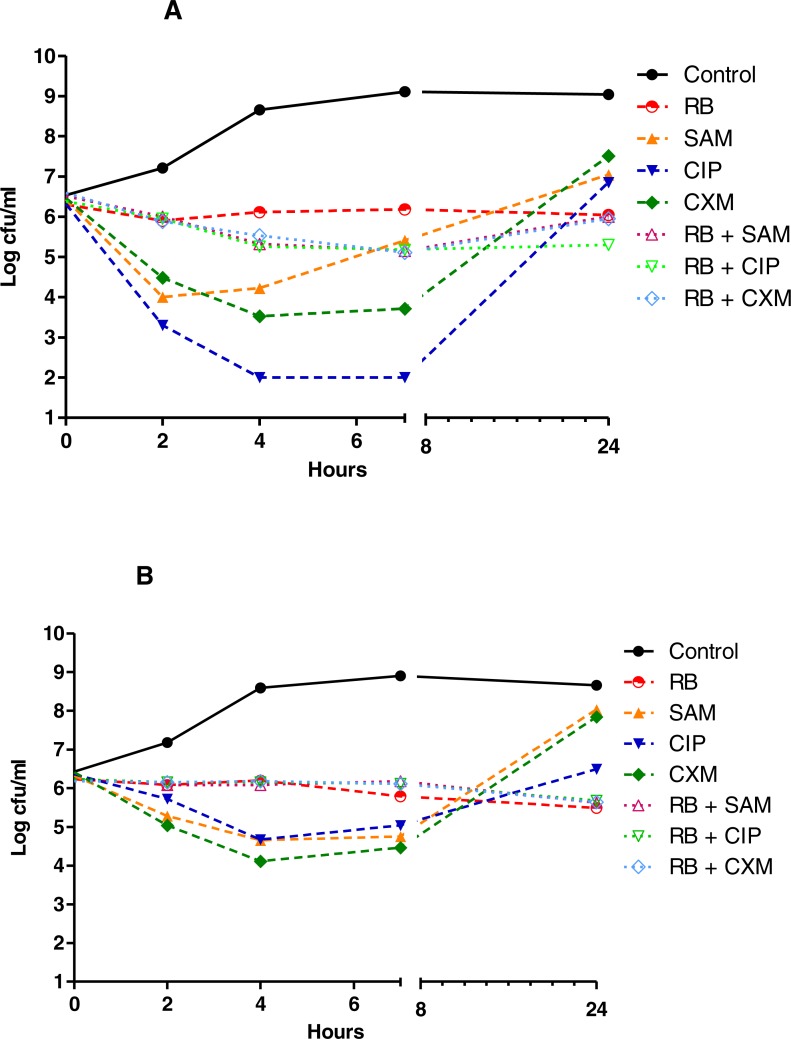
Time kill curves of herbal tea and antibiotic combinations against *E. coli* and *K. pneumoniae*. Herbal tea and antibiotic combinations observed by time-kill determinations against clinical strains of (A) *E. coli* and (B) *K. pneumonia* at 1 × MIC. The *X*- axis represents time, and *Y*-axis represents the average of logarithmic standard and clinical bacteria survivals. Control: Bacteria without any antimicrobial treatment. RB, rosehip bag; SAM, ampicillin-sulbactam; CIP, ciprofloxacin; CXM, cefuroxime.

**Figure 2:**
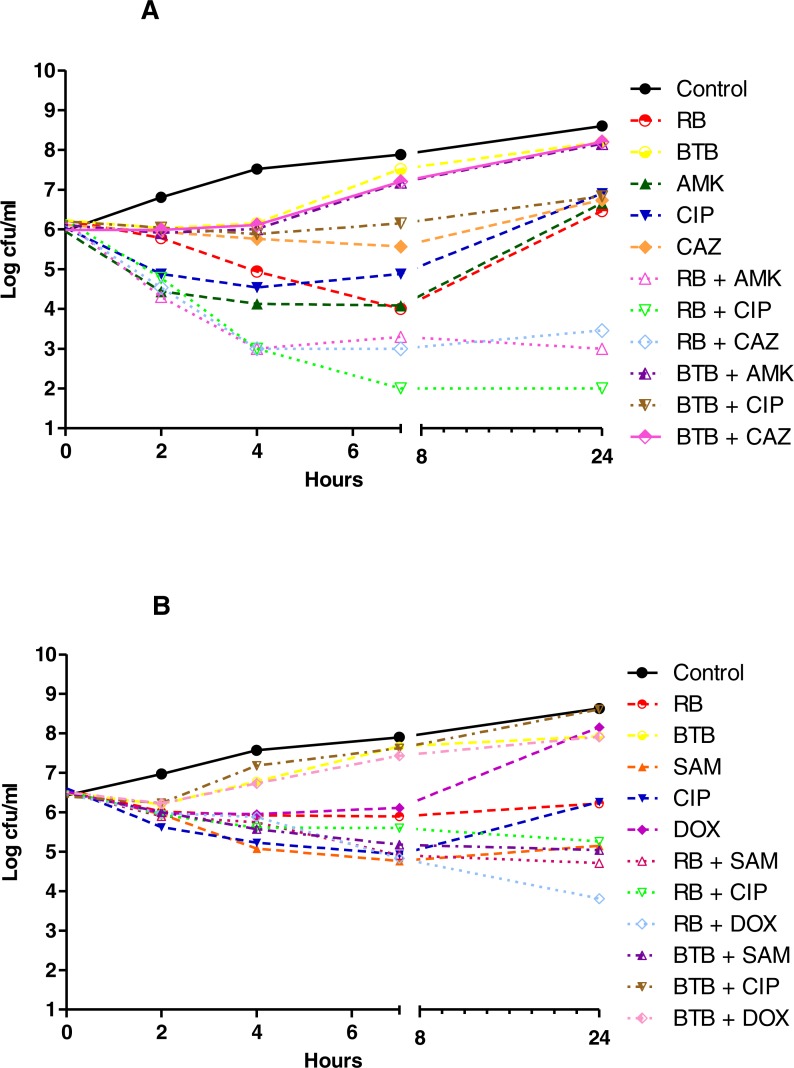
Time kill curves of herbal tea and antibiotic combinations against *P. aeruginosa* and *A. baumannii*. Herbal tea and antibiotic combinations observed by time-kill determinations against clinical strains of (A) *P. aeruginosa* and (B) *A. baumannii* at 1 × MIC. The *X*- axis represents time, and *Y*-axis represents the average of logarithmic standard and clinical bacteria survivals. Control: Bacteria without any antimicrobial treatment. RB, rosehip bag; BTB, black tea bag; AMK, amikacin; CIP, ciprofloxacin; CAZ, ceftazidime; SAM, ampicillin-sulbactam; DOX, doxycycline.

**Figure 3:**
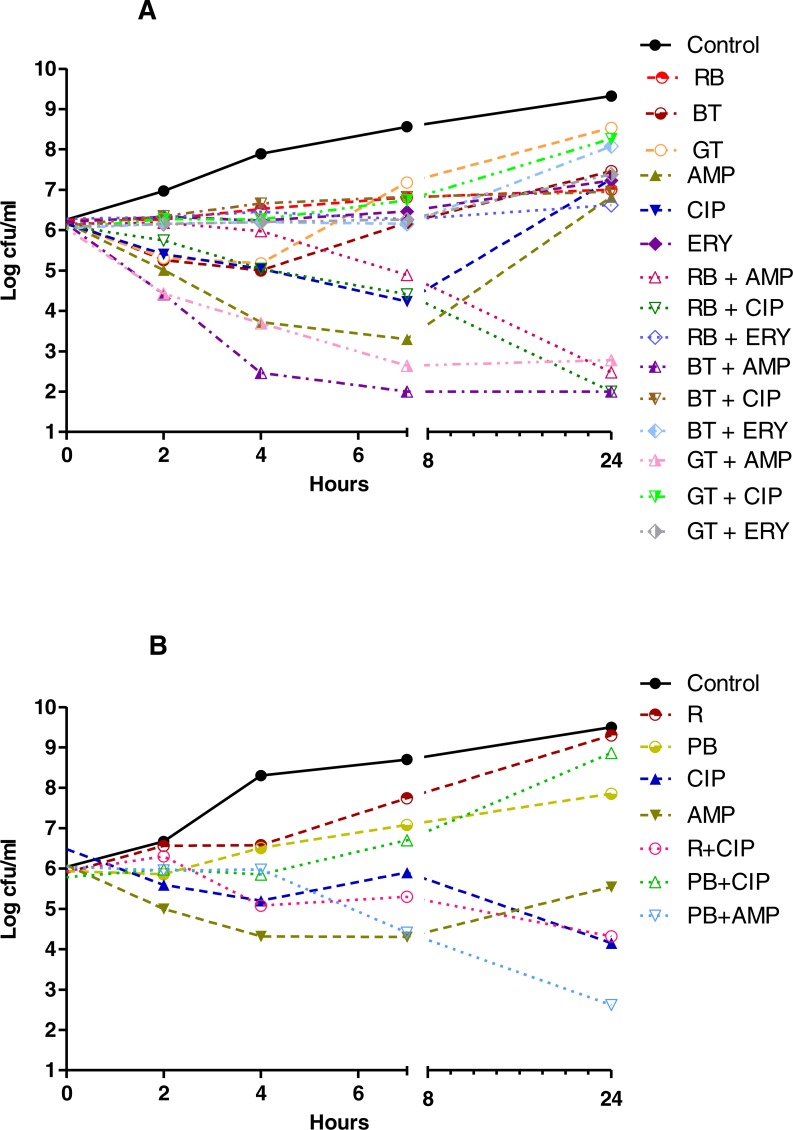
Time kill curves of herbal tea and antibiotic combinations against *S. aureus* and *E. faecalis*. Herbal tea and antibiotic combinations observed by time-kill determinations against clinical strains of (A) *S. aureus* and (B) *E. faecalis* at 1 × MIC. The *X*- axis represents time, and *Y*-axis represents the average of logarithmic standard and clinical bacteria survivals. Control: Bacteria without any antimicrobial treatment. BT, black tea; RB, rosehip bag; GT, green tea; R, rosehip; PB, pomegranate blossom; AMP, ampicillin; CIP, ciprofloxacin; ERY, erythromycin.

The results of the TKC analyses showed that with a 3 log decrease from the initial inoculum as borderline, as shown in Figs. 1–3, none of the herbal teas alone showed bactericidal activity at their indicated concentrations, whereas in the combinations with various antibiotics they were bactericidal against *P. aeruginosa* and *S. aureus*. As shown in Figs. 1–3 and Table S2, the synergistic interactions of teas and antibiotics were observed especially for rosehip bag tea and antibiotic combinations against *S. aureus* and *P. aeruginosa.* Besides this, we also observed synergistic combinations also between ampicillin and all tested tea combinations against *S. aureus.* Antagonistic or early antagonistic (4–7 h) interactions were especially observed between rosehip bag tea and antibiotics combinations against *E. coli*. Otherwise antagonistic or early antagonistic (4–7 h) interactions were rare and seen for ciprofloxacin, amikacin and cefuroxime and rosehip, black tea and green tea bag teas against several bacteria. In addition, Tables S3–S8 shows clearly the decrease or increase in the colony counts at 2, 4, 7 or 24 h.

## Discussion

Traditionally, complementary and alternative medicines are widely used and are rapidly growing health systems, including Chinese medicine, Indian ayurveda, and Arabic medicine, which use plant material, animal parts, and/or minerals (WHO, 2002). Among them, the potential health-promoting effects of plants can be traced back to the earliest recorded history (Dubick, 1986). Even though other materials such as foods are used to promote health and treat diseases, none of them have received more attention than herbs. The use of herbs includes herbal materials, herbal preparations, and finished herbal products that contain active ingredients, the parts of plants, other plant materials, or their combinations (WHO, 2002).

Some of the most popular natural products, which are gaining more importance because of their increasing antibiotic resistance, are herbal teas. Herbal teas such black, green, peppermint, sage, and thyme, are widely used for the protection and treatment of human diseases worldwide. It is known that teas, especially those that contain catechin, have many health-promoting abilities such as antibacterial, antifungal, and antiviral (Bansal et al., 2013). The antimicrobial activities of this catechin containing black and green teas has been previously demonstrated against a variety of organisms, including multiresistant clinical isolates of gram-negative and -positive bacteria and also yeasts (Anand, Kaul & Sharma, 2006; Gordon & Wareham, 2010; Hu et al., 2002; Isogai et al., 2001; Osterburg et al., 2009; Park et al., 2011). In this study, we examined the antimicrobial activities of 31 different herbal teas, both alone and in combination with chemical antimicrobials. According to these experiments, rosehip, rosehip bag, pomegranate blossom, thyme, wormwood, mint, echinacea bag, cinnamon, black, and green teas were found to be effective against most of the studied microorganisms. In general, the studied teas showed a better antimicrobial activity against gram-positive bacteria compared with the others. We hypothesized that the differences in the antimicrobial activities of the various teas would depend on either the type of microbial strain or the tea. Similar results have been obtained by other researchers (Hu et al., 2001; Novy et al., 2013). These results suggested that herbal teas could be a prophylactic or first base treatment agents for bacterial infections.

Combinations of two or more antimicrobial drugs are necessary to treat MDR or pan-resistant bacterial infections. Because mono therapy is no longer adequate, combination therapies seem to be the next logical choice; however, neither antibiotic–antibiotic combinations nor antibiotic plus non antibiotic adjuvant combinations have been successful in combating MDR infections (Tangden, 2014). Apparently, herbal teas are becoming a large part of alternative or complementary medicine, either as a single agent or as an adjuvant in antimicrobial chemotherapy (Hu et al., 2001; Cho, Oh & Oh, 2011). Antibiotic and herbal tea combinations may be recommended for severe infections in order to rapidly enhance bactericidal activity and help prevent or delay the emergence of resistance.

In this study, we examined the *in vitro* interactions between teas and antimicrobials by using one of the most simple and best known tests, namely the microbroth checkerboard technique. We have characterized all three of the expected effects, including synergistic, additive, and antagonistic interactions between the tea and antimicrobial combinations. Synergy was more frequently observed between ampicillin with almost all tea combinations against clinical MSSA strain, ampicillin–sulbactam and rosehip bag tea against *E. coli* and *A. baumanii* clinical strain, or nystatine and black tea and green tea combinations against *C. albicans* ATCC 10231. Similarly, Hu et al. (2001) found that ampicillin–sulbactam and EGCG combinations were synergistic against MRSA strains. Lee et al. (2005) also showed that ciprofloxacin and catechin combinations were synergistic against *E. coli* in a chronic bacterial prostatitis rat model. Similar results were obtained by others, particularly between catechins and antibiotic combinations against gram-positive bacteria (Hu et al., 2001; Novy et al., 2013; Zhao et al., 2002), whereas several tea combinations were tested in our study against not only gram-positive bacteria, but also gram-negative bacteria and fungi.

Although ampicillin or nystatine combinations were synergistic, most of the ciprofloxacin, erythromycin, cefuroxime, or amikacin and various tea combinations, especially with the rosehip, rosehip bag, and pomegranate blossom teas, were found to be antagonistic against almost all of the studied bacteria. Similarly Hu et al. (2002) found that EGCG showed antagonistic interactions with vancomycin, teicoplanin, or polymyxin B against MRSA.

The most desirable targets for combination therapy is synergistic drug interactions followed by the prevention of resistance and minimization of toxicity and cost. In contrast, antagonism is the most disadvantageous outcome for clinicians because the effect of the combination may be less active than that of drug alone (Pillai, Moellering Jr & Eliopoulos, 2005). In this study, we found that some antibiotic–herbal tea combinations, particularly rosehip and pomegranate blossom with ciprofloxacin or cefuroxime, have antagonistic interactions. Thus, those herbal teas should be either consumed alone or avoided in the course of the antibiotic treatment.

Although MIC is still the gold standard for determining the antimicrobial activities of agents, and the microbroth checkerboard is the most simple and widely used technique for the assessment of combination effects, these techniques do not provide any information about the time course of the antimicrobial activities. TKC studies can be used to overcome this limitation. In this study, according to the TKC results, the synergistic interactions against *S. aureus* were more frequent between ampicillin and tea combinations, just as those in the results of the checkerboard technique. On the other hand, antagonistic interactions were not as frequent in the checkerboard technique. There were only a few ciprofloxacin and tea combinations that had an antagonistic or early antagonistic (within 4–7 h) effect. The difference in our combination results between the TKC and checkerboard techniques may cause the bacteriostatic drug interactions from the checkerboard technique, whereas the bactericidal interactions were obtained from the TKC analyses. According to these results black tea, green tea and rosehip bag teas could be used effectively and safely along with ampicillin treatment as enhancer of antibacterial treatment. Conversely, these teas should be avoided during the other antibiotic treatments especially with ciprofloxacin due to their antagonistic effects.

## Conclusion

When we examined the antimicrobial activities of various herbal teas, alone and in combination with antibiotics, our findings showed that herbal teas have antimicrobial activities against gram-positive and -negative bacteria and yeast when they were used alone. The combinations of herbal teas with antibiotics showed synergistic, additive, or antagonistic effects, depending on the antibiotic or type of tea. Consequently, using herbal teas alone or with some chemical antimicrobials could be an effective alternative treatment strategy against various pathogenic microorganisms. Furthermore, herbal teas alone or in combination may help to reduce the severity of disease; however, some combinations with antibiotics could reduce the efficacy of the primary antibiotic and thus should not be used together.

##  Supplemental Information

10.7717/peerj.3467/supp-1 Table S1 Teas showing antimicrobial activity with disk diffusion assays

10.7717/peerj.3467/supp-2 Table S2 The effects of tea and antibiotic combinations against bacteria by TKC analyses Syn, Synergist; Ind, Indifference; Ant, Antagonist; RB, rosehip bag; BTB, black tea bag; BT, black tea; GT, green tea; R, rosehip; PB, pomegranate blossom; SAM, ampicillin-sulbactam; SAM, ampicillin-sulbactam; CIP, ciprofloxacin; CXM, cefuroxime; AMK, amikacin; CAZ, ceftazidime; DOX, doxycycline; AMP, ampicillin; ERY, erythromycin; (–), Not determined.

10.7717/peerj.3467/supp-3 Table S3 Average colony counts of herbal tea and antibiotic combinations in TKC analyses as log cfu/ml against *E. coli* RB, rosehip bag; SAM, ampicillin-sulbactam; CIP, ciprofloxacin; CXM, cefuroxime; *: counts were calculated as log 10 average numbers of colonies on TSA plates, considering the dilution factor.

10.7717/peerj.3467/supp-4 Table S4 Average colony counts of herbal tea and antibiotic combinations in TKC analyses as log cfu/ml against *K. pneumoniae* RB, rosehip bag; SAM, ampicillin-sulbactam; CIP, ciprofloxacin; CXM, cefuroxime; *: counts were calculated as log 10 average numbers of colonies on TSA plates, considering the dilution factor.

10.7717/peerj.3467/supp-5 Table S5 Average colony counts of herbal tea and antibiotic combinations in TKC analyses as log cfu/ml against* P. aeruginosa* RB, rosehip bag; BTB, black tea bag; AMK, amikacin; CIP, ciprofloxacin; CAZ, ceftazidime; *: counts were calculated as log 10 average numbers of colonies on TSA plates, considering the dilution factor.

10.7717/peerj.3467/supp-6 Table S6 Average colony counts of herbal tea and antibiotic combinations in TKC analyses as log cfu/ml against* A. baumanii* RB, rosehip bag; BTB, black tea bag; SAM, ampicillin-sulbactam; CIP, ciprofloxacin; DOX, doxycycline; *: counts were calculated as log 10 average numbers of colonies on TSA plates, considering the dilution factor.

10.7717/peerj.3467/supp-7 Table S7 Average colony counts of herbal tea and antibiotic combinations in TKC analyses as log cfu/ml against* S. aureus* BT, black tea; RB, rosehip bag; GT, green tea; AMP, ampicillin; CIP, ciprofloxacin; ERY, erythromycin; *: counts were calculated as log 10 average numbers of colonies on TSA plates, considering the dilution factor.

10.7717/peerj.3467/supp-8 Table S8 Average colony counts of herbal tea and antibiotic combinations in TKC analyses as log cfu/ml against *E. faecalis* R, rosehip; PB, Pomegranate blossom; CIP, ciprofloxacin; AMP, ampicillin; *: counts were calculated as log 10 average numbers of colonies on TSA plates, considering the dilution factor.

## Additional Information and Declarations

### Data Availability

The following information was supplied regarding data availability: The raw data has been supplied as a Supplemental Dataset.
