# Supplementary material for: Antimicrobial activities of widely consumed herbal teas, alone or in combination with antibiotics: an in vitro study
Source: PeerJ. 2017 Jul 26;5:e3467. doi: 10.7717/peerj.3467 (PMC5533155; doi:10.7717/peerj.3467)
Supplement: Table S1 [file peerj-05-3467-s001.docx]

|  | **Thyme** | **Wormwood** | **Mint** | **Rosehip** | **Pomegranate blossom** | **Black tea** | **Green tea** | **Oregano** | **Cinnamon** | **Rosehip bag** | **Black bag** | **Green bag** | **Sage bag** | **Mint bag** | **Echinacea bag** |
| --- | --- | --- | --- | --- | --- | --- | --- | --- | --- | --- | --- | --- | --- | --- | --- |
| **Standard strains** |  | | | | | | | | | | | | | | |
| MRSA | + | + | - | + | + | + | + | - | - | + | - | + | - | - | - |
| MSSA | - | - | - | + | + | + | + | - | - | + | - | + | - | - | - |
| *E.faecalis* | - | - | - | + | + | - | - | - | - | + | - | - | - | - | - |
| *E.coli* | - | - | - | + | + | - | - | - | - | + | - | - | - | - | - |
| *K.pneumoniae* | - | - | - | + | + | - | - | - | - | - | - | - | - | - | - |
| *P.aeruginosa* | - | - | - | + | + | - | - | - | - | + | + | - | - | - | - |
| *A.baumannii* | - | - | - | + | + | - | - | - | - | + | + | - | - | - | - |
| *C.albicans* | - | - | - | - | - | + | + | - | - | - | - | - | - | - | - |
| **Clinical isolates** |  | | | | | | | | | | | | | | |
| MRSA | + | + | + | + | + | + | + | + | - | + | + | + | + | + | - |
| MSSA | + | + | + | + | + | + | + | + | - | + | + | + | + | + | + |
| *E.faecalis* | - | - | - | + | + | - | - | - | - | + | - | - | - | - | - |
| *E.coli* | - | - | - | + | + | - | - | - | - | + | - | - | - | - | - |
| *K.pneumoniae* | - | - | - | + | + | - | - | - | - | + | - | - | - | - | - |
| *P.aeruginosa* | - | - | - | + | + | - | + | - | - | + | + | - | - | - | - |
| *A.baumannii* | - | - | - | + | + | - | + | - | - | + | + | + | - | - | - |
| *C.albicans* | - | - | - | - | - | - | - | - | + | - | - | - | - | - | - |
