# Supplementary material for: Antimicrobial activities of widely consumed herbal teas, alone or in combination with antibiotics: an in vitro study
Source: PeerJ. 2017 Jul 26;5:e3467. doi: 10.7717/peerj.3467 (PMC5533155; doi:10.7717/peerj.3467)
Supplement: Table S2 — Syn, Synergist; Ind, Indifference; Ant, Antagonist; RB, rosehip bag; BTB, black tea bag; BT, black tea; GT, green tea; R, rosehip; PB, pomegranate blossom; SAM, ampicillin-sulbactam; SAM, ampicillin-sulbactam; CIP, ciprofloxacin; CXM, cefuroxime; AMK, amikacin; CAZ, ceftazidime; DOX, doxycycline; AMP, ampicillin; ERY, erythromycin; (–), Not determined. [file peerj-05-3467-s002.docx]

| **Combinations** | ***E. coli*** | ***K. pneumoniae*** | ***P. aeruginosa*** | ***A. baumanii*** | ***S. aureus*** | ***E. faecalis*** |
| --- | --- | --- | --- | --- | --- | --- |
| RB + SAM | Ind | Ind | - | Ind | - | - |
| RB + CIP | Ind | Ind | Syn | Ind | Syn | - |
| RB + CXM | Ind | Ind | - | - | - | - |
| RB + AMK | - | - | Syn | - | - | - |
| RB + CAZ | - | - | Syn | - | - | - |
| RB + DOX | - | - | - | Syn | - | - |
| RB + AMP | - | - | - | - | Syn | - |
| RB + ERY | - | - | - | - | Ind | - |
| BTB + SAM | - | - | - | Ind | - | - |
| BTB + AMK | - | - | Ant | - | - | - |
| BTB + CIP | - | - | Ind | Ant | - | - |
| BTB + CAZ | - | - | Ind | - | - | - |
| BTB + DOX | - | - | - | Ind | - | - |
| BT + AMP | - | - | - | - | Syn | - |
| BT + CIP | - | - | - | - | Ind | - |
| BT + ERY | - | - | - | - | Ind | - |
| GT + AMP | - | - | - | - | Syn | - |
| GT + CIP | - | - | - | - | Ind | - |
| GT + ERY | - | - | - | - | Ind | - |
| R + CIP | - | - | - | - | - | Ind |
| PB + CIP | - | - | - | - | - | Ant |
| PB + AMP | - | - | - | - | - | Syn |
