# Supplementary material for: Antimicrobial activities of widely consumed herbal teas, alone or in combination with antibiotics: an in vitro study
Source: PeerJ. 2017 Jul 26;5:e3467. doi: 10.7717/peerj.3467 (PMC5533155; doi:10.7717/peerj.3467)
Supplement: Table S3 — RB, rosehip bag; SAM, ampicillin-sulbactam; CIP, ciprofloxacin; CXM, cefuroxime; *: counts were calculated as log 10 average numbers of colonies on TSA plates, considering the dilution factor. [file peerj-05-3467-s003.docx]

|  | **Average colony counts (log cfu/ml)*** | | | | | | | |
| --- | --- | --- | --- | --- | --- | --- | --- | --- |
| **Hours** | **Control** | **RB** | **SAM** | **CIP** | **CXM** | **RB+SAM** | **RB+CIP** | **RB+CXM** |
| 0. | 6,54 | 6,30 | 6,43 | 6,32 | 6,46 | 6,51 | 6,38 | 6,59 |
| 2. | 7,21 | 5,90 | 4,00 | 3,30 | 4,48 | 6,00 | 5,95 | 5,88 |
| 4. | 8,66 | 6,11 | 4,22 | 2,00 | 3,52 | 5,32 | 5,26 | 5,53 |
| 7. | 9,11 | 6,18 | 5,41 | 2,00 | 3,71 | 5,15 | 5,18 | 5,11 |
| 24. | 9,04 | 6,04 | 7,04 | 6,85 | 7,51 | 6,00 | 5,30 | 5,95 |
