# Supplementary material for: Antimicrobial activities of widely consumed herbal teas, alone or in combination with antibiotics: an in vitro study
Source: PeerJ. 2017 Jul 26;5:e3467. doi: 10.7717/peerj.3467 (PMC5533155; doi:10.7717/peerj.3467)
Supplement: Table S4 — RB, rosehip bag; SAM, ampicillin-sulbactam; CIP, ciprofloxacin; CXM, cefuroxime; *: counts were calculated as log 10 average numbers of colonies on TSA plates, considering the dilution factor. [file peerj-05-3467-s004.docx]

|  | **Average colony counts (log cfu/ml)*** | | | | | | | |
| --- | --- | --- | --- | --- | --- | --- | --- | --- |
| **Hours** | **Control** | **RB** | **SAM** | **CIP** | **CXM** | **RB+SAM** | **RB+CIP** | **RB+CXM** |
| 0. | 6,43 | 6,23 | 6,32 | 6,36 | 6,41 | 6,23 | 6,23 | 6,18 |
| 2. | 7,18 | 6,08 | 5,28 | 5,72 | 5,04 | 6,08 | 6,15 | 6,15 |
| 4. | 8,59 | 6,20 | 4,65 | 4,67 | 4,11 | 6,08 | 6,15 | 6,18 |
| 7. | 8,90 | 5,79 | 4,75 | 5,04 | 4,46 | 6,18 | 6,11 | 6,11 |
| 24. | 8,66 | 5,49 | 8,03 | 6,49 | 7,84 | 5,62 | 5,68 | 5,64 |
