# Supplementary material for: Antimicrobial activities of widely consumed herbal teas, alone or in combination with antibiotics: an in vitro study
Source: PeerJ. 2017 Jul 26;5:e3467. doi: 10.7717/peerj.3467 (PMC5533155; doi:10.7717/peerj.3467)
Supplement: Table S5 — RB, rosehip bag; BTB, black tea bag; AMK, amikacin; CIP, ciprofloxacin; CAZ, ceftazidime; *: counts were calculated as log 10 average numbers of colonies on TSA plates, considering the dilution factor. [file peerj-05-3467-s005.docx]

|  | **Average colony counts (log cfu/ml)*** | | | | | | | | | | | |
| --- | --- | --- | --- | --- | --- | --- | --- | --- | --- | --- | --- | --- |
| **Hours** | **Control** | **RB** | **BTB** | **AMK** | **CIP** | **CAZ** | **RB+AMK** | **RB+CIP** | **RB+CAZ** | **BTB+AMK** | **BTB+CIP** | **BTB+CAZ** |
| 0. | 5,97 | 6,23 | 6,23 | 5,95 | 6,04 | 6,11 | 6,18 | 6,23 | 6,11 | 6,11 | 6,20 | 5,99 |
| 2. | 6,81 | 5,78 | 6,03 | 4,44 | 4,88 | 5,94 | 4,30 | 4,78 | 4,54 | 5,91 | 6,04 | 5,99 |
| 4. | 7,52 | 4,94 | 6,15 | 4,13 | 4,54 | 5,76 | 3,00 | 3,00 | 3,00 | 6,01 | 5,88 | 6,11 |
| 7. | 7,88 | 4,00 | 7,52 | 4,08 | 4,88 | 5,57 | 3,30 | 2,00 | 3,00 | 7,18 | 6,15 | 7,20 |
| 24. | 8,60 | 6,46 | 8,20 | 6,66 | 6,90 | 6,74 | 3,00 | 2,00 | 3,46 | 8,15 | 6,83 | 8,20 |
