# Supplementary material for: Antimicrobial activities of widely consumed herbal teas, alone or in combination with antibiotics: an in vitro study
Source: PeerJ. 2017 Jul 26;5:e3467. doi: 10.7717/peerj.3467 (PMC5533155; doi:10.7717/peerj.3467)
Supplement: Table S6 — RB, rosehip bag; BTB, black tea bag; SAM, ampicillin-sulbactam; CIP, ciprofloxacin; DOX, doxycycline; *: counts were calculated as log 10 average numbers of colonies on TSA plates, considering the dilution factor. [file peerj-05-3467-s006.docx]

|  | **Average colony counts (log cfu/ml)*** | | | | | | | | | | | |
| --- | --- | --- | --- | --- | --- | --- | --- | --- | --- | --- | --- | --- |
| **Hours** | **Control** | **RB** | **BTB** | **SAM** | **CIP** | **DOX** | **RB+SAM** | **RB+CIP** | **RB+DOX** | **BTB+SAM** | **BTB+CIP** | **BTB+DOX** |
| 0. | 5,97 | 6,56 | 6,49 | 6,53 | 6,60 | 6,52 | 6,49 | 6,53 | 6,45 | 6,49 | 6,40 | 6,51 |
| 2. | 6,81 | 6,02 | 6,20 | 5,95 | 5,62 | 5,97 | 5,90 | 5,93 | 5,97 | 6,00 | 6,23 | 6,22 |
| 4. | 7,52 | 5,92 | 6,78 | 5,08 | 5,23 | 5,95 | 5,74 | 5,61 | 5,88 | 5,57 | 7,18 | 6,73 |
| 7. | 7,88 | 5,89 | 7,67 | 4,76 | 4,93 | 6,11 | 4,91 | 5,60 | 4,83 | 5,18 | 7,62 | 7,43 |
| 24. | 8,60 | 6,22 | 7,92 | 5,15 | 6,26 | 8,15 | 4,71 | 5,26 | 3,81 | 5,04 | 8,61 | 7,91 |
