# Supplementary material for: Antimicrobial activities of widely consumed herbal teas, alone or in combination with antibiotics: an in vitro study
Source: PeerJ. 2017 Jul 26;5:e3467. doi: 10.7717/peerj.3467 (PMC5533155; doi:10.7717/peerj.3467)
Supplement: Table S7 — BT, black tea; RB, rosehip bag; GT, green tea; AMP, ampicillin; CIP, ciprofloxacin; ERY, erythromycin; *: counts were calculated as log 10 average numbers of colonies on TSA plates, considering the dilution factor. [file peerj-05-3467-s007.docx]

|  | **Average colony counts (log cfu/ml)*** | | | | | | | | | | | | | | | |
| --- | --- | --- | --- | --- | --- | --- | --- | --- | --- | --- | --- | --- | --- | --- | --- | --- |
| **Hours** | **Control** | **BT** | **RB** | **GT** | **AMP** | **CIP** | **ERY** | **BT+AMP** | **BT+CIP** | **BT+ERY** | **RB+AMP** | **RB+CIP** | **RB+ERY** | **GT+AMP** | **GT+CIP** | **GT+ERY** |
| 0. | 6,26 | 6,23 | 6,26 | 6,15 | 6,18 | 6,15 | 6,20 | 6,15 | 6,11 | 6,04 | 6,15 | 6,11 | 6,28 | 6,04 | 6,04 | 6,08 |
| 2. | 6,97 | 5,26 | 6,26 | 5,31 | 5,01 | 5,40 | 6,33 | 4,41 | 6,34 | 6,15 | 6,18 | 5,74 | 6,32 | 4,43 | 6,27 | 6,15 |
| 4. | 7,89 | 5,00 | 6,52 | 5,18 | 3,72 | 5,04 | 6,23 | 2,46 | 6,66 | 6,20 | 5,97 | 5,04 | 6,45 | 3,69 | 6,26 | 6,20 |
| 7. | 8,56 | 6,18 | 6,81 | 7,18 | 3,30 | 4,23 | 6,46 | 2,00 | 6,82 | 6,15 | 4,89 | 4,41 | 6,26 | 2,64 | 6,74 | 6,26 |
| 24. | 9,32 | 7,46 | 7,00 | 8,53 | 6,82 | 7,26 | 7,23 | 2,00 | 6,95 | 8,08 | 2,48 | 2,00 | 6,62 | 2,78 | 8,26 | 7,38 |
