# Supplementary material for: Antimicrobial activities of widely consumed herbal teas, alone or in combination with antibiotics: an in vitro study
Source: PeerJ. 2017 Jul 26;5:e3467. doi: 10.7717/peerj.3467 (PMC5533155; doi:10.7717/peerj.3467)
Supplement: Table S8 — R, rosehip; PB, Pomegranate blossom; CIP, ciprofloxacin; AMP, ampicillin; *: counts were calculated as log 10 average numbers of colonies on TSA plates, considering the dilution factor. [file peerj-05-3467-s008.docx]

|  | **Average colony counts (log cfu/ml)*** | | | | | | | |
| --- | --- | --- | --- | --- | --- | --- | --- | --- |
| **Hours** | **Control** | **R** | **PB** | **CIP** | **AMP** | **R+CIP** | **PB+CIP** | **PB+AMP** |
| 0. | 6,04 | 5,90 | 5,93 | 6,48 | 6,08 | 5,93 | 5,78 | 6,04 |
| 2. | 6,67 | 6,56 | 5,86 | 5,59 | 5,00 | 6,30 | 5,98 | 5,95 |
| 4. | 8,30 | 6,58 | 6,51 | 5,20 | 4,32 | 5,08 | 5,85 | 5,97 |
| 7. | 8,70 | 7,74 | 7,08 | 5,90 | 4,30 | 5,30 | 6,70 | 4,41 |
| 24. | 9,50 | 9,30 | 7,85 | 4,15 | 5,54 | 4,32 | 8,86 | 2,62 |
